# Macroscopic versus rapid on-site evaluation for EUS-guided tissue acquisition of solid pancreatic masses: a systematic review, diagnostic test accuracy meta-analysis, and network meta-analysis

**DOI:** 10.3389/fonc.2026.1943706

**Published:** 2026-10-19

**Authors:** Huifang Xu, Jiongming He, Gong Chen, Biao Chen

**Affiliations:** 1 Medical Ultrasound Department, The Second Hospital of Wuhan Iron and Steel (Group) Corp, Wuhan, China; 2 Department of Thoracic and Cardiovascular Surgery, Third Hospital of Wuhan City, Wuhan, China; 3 Cancer Center, Renmin Hospital of Wuhan University, Wuhan, China

**Keywords:** diagnostic test accuracy, endoscopic ultrasound, fine-needle aspiration, fine-needle biopsy, macroscopic on-site evaluation, network meta-analysis, pancreatic mass, rapid on-site evaluation

## Abstract

**Background:**

Endoscopic ultrasound-guided tissue acquisition (EUS-TA) is central to the pathological diagnosis of solid pancreatic masses, but the comparative value of rapid on-site cytologic evaluation (ROSE), macroscopic on-site evaluation (MOSE), and no on-site evaluation (NONE) may vary by needle type and remains uncertain.

**Methods:**

We performed a PRISMA-DTA systematic review registered in PROSPERO (CRD420251241446). Potential cohort overlap and data dependency were assessed before quantitative synthesis. Diagnostic 2 × 2 data were synthesized with a bivariate binomial-normal random-effects model. Independent direct comparisons were pooled by random-effects meta-analysis for correct versus incorrect diagnosis, with complementary absolute differences in sensitivity and specificity. An exploratory three-node network meta-analysis (NMA) compared ROSE, MOSE, and NONE.

**Results:**

Thirty-one eligible reports contributed 67 strategy-specific diagnostic datasets. After excluding 21 duplicate, nested, paired, or otherwise dependent datasets, 46 independent datasets representing 4,901 arm-level observations were included in the primary DTA. Pooled sensitivity was 0.94 (95% CI 0.92–0.95) and specificity was 1.00 (0.98–1.00); Deeks’ asymmetry test was positive (P <0.001). In direct comparisons, ROSE improved composite diagnostic accuracy versus NONE in FNA studies (OR 5.56, 95% CI 1.76–17.62; k = 4), but not in FNB studies (OR 1.08, 0.42–2.79; k = 4); the FNA-versus-FNB interaction was exploratory (P = 0.012). MOSE versus NONE in FNB was uncertain (OR 1.31, 0.60–2.87; k = 4), as was MOSE versus ROSE (OR 1.53, 0.05–49.13; k = 3). The exploratory NMA did not identify a statistically clear difference for ROSE versus NONE (OR 1.67, 0.94–2.95), MOSE versus NONE (OR 1.89, 0.86–4.20), or MOSE versus ROSE (OR 1.14, 0.48–2.71).

**Conclusions:**

EUS-TA showed high overall diagnostic performance. ROSE may add value in FNA-era workflows, whereas a clear incremental benefit was not detected with FNB. MOSE remains a plausible resource-sparing strategy, but comparative estimates are imprecise. The NMA should be regarded as hypothesis-generating because of sparse comparisons, methodological heterogeneity, small-study effects, and low-to-very-low certainty.

**Systematic review registration:**

https://www.crd.york.ac.uk/PROSPERO/, identifier CRD420251241446.

## 1 Introduction

Endoscopic ultrasound-guided tissue acquisition (EUS-TA) is a routine gateway to pathological confirmation and treatment planning for solid pancreatic masses (1, 2). Fine-needle aspiration (FNA) historically relied on cytologic material and often benefited from immediate specimen feedback, whereas modern fine-needle biopsy (FNB) needles are designed to acquire larger histologic cores and may reduce dependence on on-site cytology (3–8). The improved tissue architecture of FNB is also relevant to immunohistochemistry and molecular profiling, for which pre-analytic specimen quality is increasingly important (9, 10). This evolution from FNA toward FNB has changed the clinical question from whether on-site assessment can improve sampling in general to whether its incremental value persists when contemporary tissue-acquisition technology is used.

Rapid on-site evaluation (ROSE) provides immediate cytologic feedback on adequacy and may guide the number of passes, but it requires trained cytopathology personnel and coordination in the endoscopy suite (7, 11, 12). Macroscopic on-site evaluation (MOSE) attempts to provide a simpler alternative by judging visible tissue cores or specimen characteristics without formal real-time cytology. Visible-core length, formal macroscopic criteria, and randomized MOSE protocols have all been evaluated as ways to make this assessment more reproducible (13–15). Both strategies are operationally attractive for different settings, yet their definitions, thresholds, operator training, number-of-pass strategies, and relationships to needle design vary substantially across studies (13–17).

Existing evidence is not uniform. A prior meta-analysis of EUS-FNB found that ROSE was associated with higher diagnostic accuracy overall, but the apparent benefit was not observed when analyses were restricted to newer end-cutting needles (18). This finding is clinically important because it implies that the value of on-site evaluation may be conditional on the sampling platform rather than a universal property of ROSE itself. Recent evidence comparing FNA with FNB and randomized ROSE with no-ROSE workflows likewise emphasizes the influence of needle technology and study design on diagnostic yield and procedural efficiency (6, 12, 17). In addition, direct comparisons among ROSE, MOSE, and no on-site evaluation (NONE) are sparse, and some publications report repeated assessments, nested subsets, preliminary abstracts, or overlapping cohorts. Treating such records as independent can produce artificially narrow confidence intervals and can materially alter a network meta-analysis.

This review addressed two complementary questions: how accurately EUS-guided tissue acquisition diagnoses solid pancreatic masses, and whether one on-site evaluation strategy improves diagnostic accuracy over another within a comparable sampling platform. We therefore combined DTA meta-analysis with direct comparative meta-analysis and an exploratory network meta-analysis of ROSE, MOSE, and NONE. Potentially overlapping or dependent datasets were assessed before quantitative synthesis, direct comparisons were separated by FNA and FNB where appropriate, and composite diagnostic-accuracy findings were interpreted together with sensitivity and specificity.

## 2 Methods

### 2.1 Protocol, registration, and reporting

The review was registered in PROSPERO (CRD420251241446) and is reported in accordance with PRISMA 2020 and PRISMA-DTA (19, 20). A completed PRISMA 2020 checklist is provided in **Supplementary Table S1**. The registered protocol primarily emphasized FNB. Because eligible studies using FNA and mixed EUS-TA workflows were identified during study selection, the final eligibility framework encompassed EUS-guided tissue acquisition more broadly; this post-registration protocol amendment is reported transparently. Comparative analyses, including the NMA, were considered exploratory.

### 2.2 Eligibility criteria

We included studies of adults undergoing EUS-guided sampling of suspected solid pancreatic lesions when at least one eligible on-site strategy (ROSE, MOSE, or NONE) was evaluated and a 2 × 2 diagnostic table could be extracted or reconstructed. Randomized trials, prospective comparative studies, retrospective comparative studies, and eligible diagnostic cohorts were considered. FNA, FNB, and mixed/unclear FNA-FNB workflows were retained but coded separately. Studies confined to cystic lesions, nonpancreatic targets without separable pancreatic data, case reports, narrative reviews, and reports without extractable diagnostic outcomes were excluded.

### 2.3 Search strategy and study selection

PubMed, Embase, the Cochrane Central Register of Controlled Trials, and WanFang were searched through 15 December 2025 using controlled vocabulary and free-text terms for pancreatic lesions, EUS-guided tissue acquisition, FNA/FNB, ROSE, MOSE, macroscopic visible core assessment, and on-site evaluation. The full database-specific electronic search strategies are provided in **Supplementary Table S2**. Registry and citation searching supplemented the database search. Two reviewers screened records and assessed potentially eligible reports.

### 2.4 Data extraction and overlap/dependency audit

For each analyzable strategy-specific dataset, we extracted study identifier, author, year, sampling method, intervention strategy, analyzable sample size, and true-positive (TP), false-positive (FP), false-negative (FN), and true-negative (TN) counts. The 46 datasets included in the primary DTA are listed in **Supplementary Table S3**. Before quantitative synthesis, all 67 extracted datasets underwent a structured assessment for duplicate publication, overlapping recruitment, nested subsets, repeated diagnostic endpoints, and paired within-person assessments. Exact duplicate 2 × 2 tables and preliminary reports superseded by fuller publications were not treated as independent. When two reports appeared to derive from the same registry or recruitment frame, the later or more complete source was preferentially retained. Paired or repeated diagnostic assessments were excluded from independent synthesis when the within-person covariance required for a valid paired effect estimate was unavailable.

### 2.5 Risk of bias and certainty of evidence

Risk of bias was assessed with QUADAS-2 across patient selection, index test, reference standard, and flow/timing domains (21). Particular attention was paid to nonconsecutive enrollment, unclear blinding, *post-hoc* specimen thresholds, and incomplete reference-standard verification. Certainty was graded separately for overall DTA and the direct and network comparative questions, with ratings summarized in **Supplementary Table S4**. The complete 67-row overlap/dependency assessment and all retain/exclude decisions are documented in **Supplementary Table S5**. Considering methodological heterogeneity, sparse comparative evidence, the positive Deeks’ asymmetry test, and indirectness across needle generations, certainty was rated low for overall DTA and the better-populated direct comparisons and very low for the sparsest MOSE and NMA contrasts.

### 2.6 Diagnostic test accuracy synthesis

Primary DTA synthesis used the 46 datasets retained after the overlap/dependency audit. Sensitivity and specificity were modeled jointly with a bivariate binomial-normal random-effects model, which accounts for their within-study relationship and avoids applying continuity corrections to the likelihood solely because a study reports a zero cell (22, 23). Summary estimates with 95% confidence intervals were obtained for the overall dataset and descriptively for ROSE, MOSE, and NONE. Between-dataset heterogeneity is reported using the random-effects standard deviations on the logit sensitivity and specificity scales rather than relying on a single conventional I² statistic, which is not well suited to a bivariate DTA model.

### 2.7 Direct comparative meta-analysis and sensitivity/specificity differences

Independent within-study contrasts were analyzed for correct versus incorrect diagnosis. To reduce clinical transitivity violations, ROSE versus NONE was analyzed separately for FNA and FNB, whereas MOSE comparisons were analyzed within FNB when the sampling class was clear. The independent study-level contrasts are listed in **Supplementary Table S6**. Pairwise effects were pooled with random-effects models using restricted maximum likelihood (REML) for between-study variance and modified Hartung–Knapp small-sample inference (variance scale not allowed below 1), with t-based 95% confidence intervals. For clinical interpretability, we also meta-analyzed absolute within-study differences in sensitivity and specificity using the same REML/modified Hartung–Knapp framework. The FNA-versus-FNB interaction for ROSE versus NONE was treated as exploratory effect modification rather than a definitive subgroup claim.

### 2.8 Exploratory network meta-analysis and inconsistency

An exploratory three-node NMA connected ROSE, MOSE, and NONE using the composite endpoint of correct versus incorrect diagnosis. A frequentist random-effects consistency model was fitted on the log-OR scale with a common REML heterogeneity variance across network contrasts. Treatment effects were estimated by inverse-variance weighted network meta-regression with NONE as the reference node. Local inconsistency in the closed three-treatment loop was assessed as the difference between the direct MOSE-versus-ROSE log-OR and the corresponding indirect estimate through NONE (inconsistency factor). Because the network is sparse and the composite endpoint can conceal sensitivity/specificity tradeoffs, network estimates were not allowed to supersede the direct DTA evidence.

### 2.9 Small-study effects, software, and analysis hierarchy

Deeks’ regression asymmetry test was applied to the primary DTA dataset using the conventional inverse-square-root effective-sample-size regression (24). Analyses were performed in Python 3 using SciPy, statsmodels, and explicit REML meta-analytic routines; DTA was fitted with a binomial-normal bivariate model using Gauss–Hermite quadrature. The dedicated statsmodels meta-analysis implementation was used as an independent sensitivity check, while reported pairwise estimates follow the REML/modified Hartung–Knapp specification described above. The Stata metadta framework was used as a methodological cross-check for DTA modeling (25). The analysis hierarchy was: (1) bivariate DTA of independent datasets; (2) independent direct comparisons, including sensitivity/specificity differences; and (3) exploratory NMA. Supportive restricted analyses are presented in randomized-comparison-only direct meta-analysis and explicitly standardized MOSE analyses.

## 3 Results

### 3.1 Study selection and evidence base

The search identified 578 records across databases, registers, citation searching, and other sources. After screening and full-text assessment, 31 reports met the eligibility criteria (15, 26–55). These reports contributed 67 candidate strategy-specific diagnostic datasets. The study-selection process is shown in **Figure 1**. The PRISMA checklist and full electronic search strategies are provided in **Supplementary Tables S1**, **S2**.

**Figure 1:**
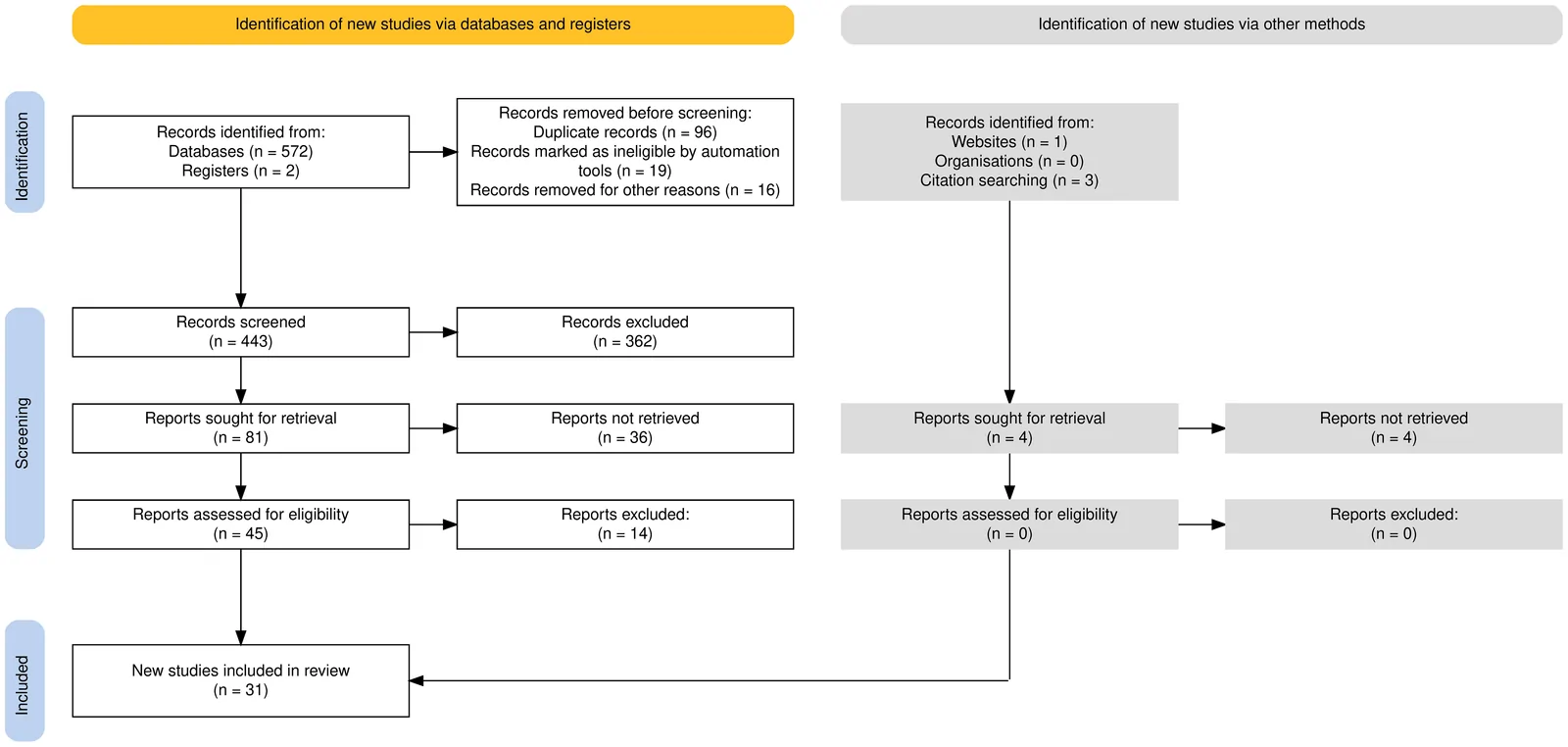
PRISMA 2020 flow diagram of the literature search and study selection process. The diagram details the systematic identification, screening, eligibility assessment, and inclusion of studies for the systematic review and network meta-analysis.

### 3.2 Cohort-overlap and dependency audit

The audit identified 21 datasets that could not be regarded as statistically independent for the primary synthesis. These included exact or likely duplicate publications/cohorts (nine datasets), alternative dependent endpoints or nested subsets (five datasets), and paired/repeated assessments for which within-person covariance was not available (seven datasets). Examples included the exact 2 × 2 duplication between the Li and Zhao data, the preliminary Mangiavillano abstract superseded by the full randomized trial, overlapping registry reports from the Lariño/Iglesias group, repeated Naidu endpoints, the Okasha nested subset, and paired/repeated Sundaram and Wong assessments. Forty-six datasets representing 4,901 arm-level observations remained for primary DTA (**Table 1**, **Supplementary Table S3**). These are arm-level analytic observations, not a claim of 4,901 unique patients across every publication.

**Table 1:** Characteristics of report-level source groups contributing independent diagnostic data after the overlap/dependency audit.

Author	Year	Retained strategy	Sampling method	Datasets	Arm-level n
Amalou	2025	MOSE / NONE	FNB	2	148
Cai	2021	ROSE / NONE	FNA	2	87
Cai	2024	ROSE / NONE	EUS-TA / not fully specified	2	137
Chong, C. C. N.	2020	MOSE / NONE	EUS-TA / not fully specified	2	141
Comas	2024	MOSE / ROSE	FNB	2	132
Crinò	2021	ROSE / NONE	FNB	2	771
Dbouk	2025	ROSE / NONE	FNB	2	230
De Lusong	2024	ROSE / NONE	FNB	2	78
Fabbri	2017	ROSE / NONE	FNB	2	333
Gaia	2022	MOSE / NONE	FNB	2	76
Iglesias-Garcia	2024	ROSE / NONE	Mixed/unclear FNA-FNB	2	566
Ishikawa	2022	MOSE / ROSE	FNB	2	120
Li	2023	ROSE / NONE	FNA	3	188
Lin	2023	ROSE / NONE	FNA	3	168
Mangiavillano, B.	2023	MOSE / NONE	FNB	2	370
Naidu	2021	ROSE / NONE	Mixed/unclear FNA-FNB	2	210
Ogura	2024	ROSE / MOSE	FNB	2	171
Okasha	2024	MOSE	Mixed/unclear FNA-FNB	1	215
Ratnaraj	2018	ROSE / NONE	Mixed/unclear FNA-FNB	2	273
So	2021	MOSE	FNB	1	74
Sonthalia, N.	2024	MOSE / NONE	FNB	2	96
Xu	2024	NONE	FNB	2	193
Zhang	2020	ROSE / NONE	FNA	2	124

Arm-level n is an analytic count and is not interpreted as a unique-patient count across all publications. Only datasets retained after the cohort-overlap/dependency audit are shown. So et al. (2021) contributed a single MOSE diagnostic dataset to the overall DTA; no eligible independent comparator arm was available in the extracted data, so this report did not contribute to the direct pairwise or NMA estimates.

### 3.3 Methodological quality assessment

QUADAS-2 judgments for the 31 eligible reports are shown in **Figure 2**. Study quality was heterogeneous: several reports were at low risk across domains, whereas others raised concerns because consecutive enrollment, blinding, prespecification of diagnostic thresholds, or complete reference-standard verification was unclear. A minority had high-risk judgments related to patient flow or reference-standard adjudication. These limitations, together with the positive Deeks’ asymmetry signal and between-study methodological heterogeneity, supported low or very-low certainty ratings (**Supplementary Table S4**). The complete 67-row overlap/dependency assessment is provided in **Supplementary Table S5**.

**Figure 2:**
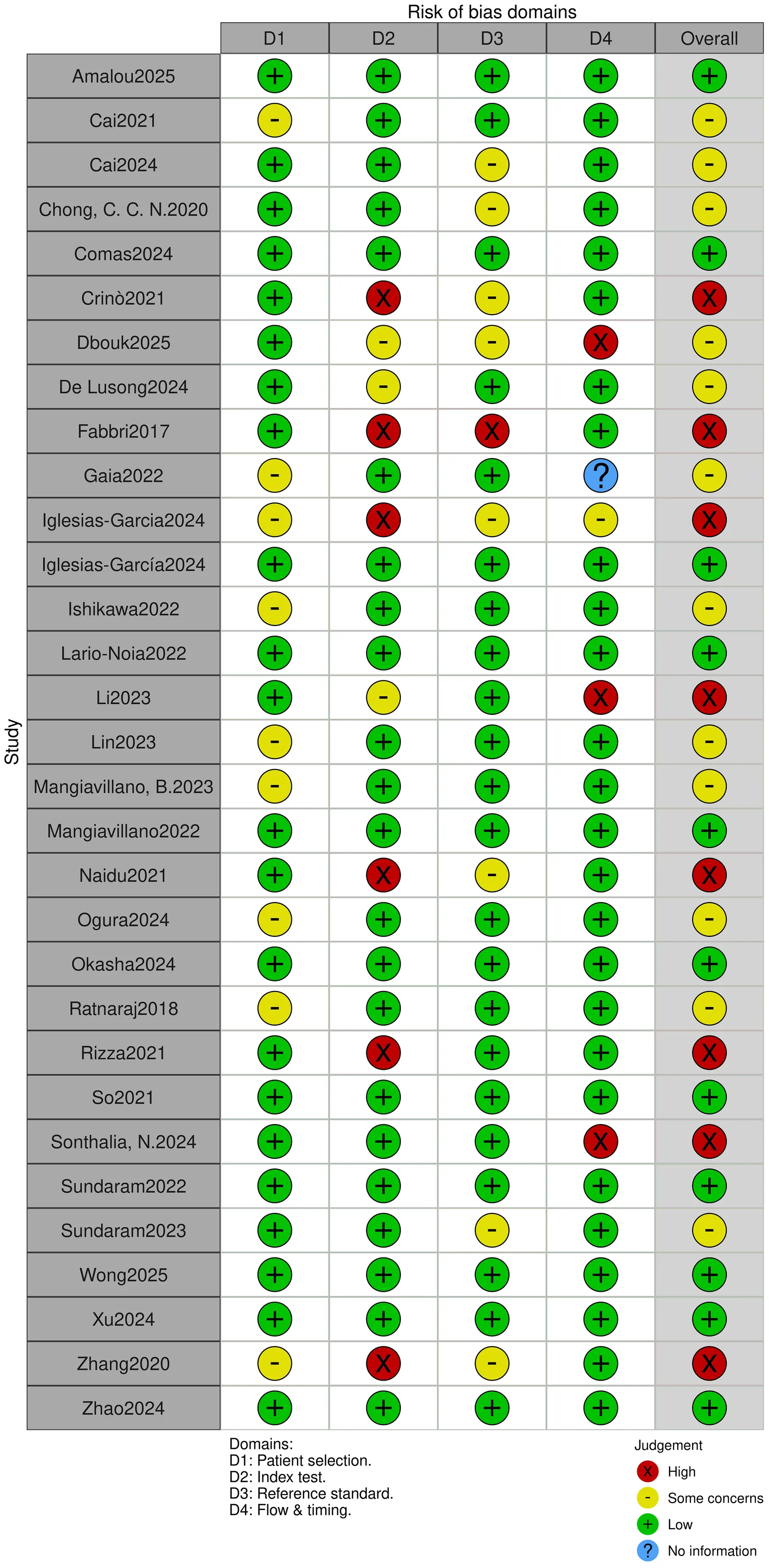
Methodological quality and risk-of-bias assessment of the included studies based on the QUADAS-2 tool. Green circles indicate low risk, yellow circles indicate unclear risk/some concerns, and red circles indicate high risk of bias.

### 3.4 Overall diagnostic performance

Across the 46 independent diagnostic datasets, there were 3,974 TP, 313 FN, 24 FP, and 590 TN classifications. Bivariate pooled sensitivity was 0.94 (95% CI 0.92–0.95) and pooled specificity was 1.00 (95% CI 0.98–1.00) (**Table 2**, **Figures 3**, **4**, **Supplementary Figure S1**). The corresponding random-effects standard deviations were 0.88 on the logit-sensitivity scale and 3.87 on the logit-specificity scale, indicating material between-dataset heterogeneity, particularly in the sparse false-positive component. Strategy-specific descriptive estimates were: ROSE sensitivity 0.96 (0.94–0.97) and specificity 1.00 (0.94–1.00); MOSE sensitivity 0.94 (0.91–0.96) and specificity 1.00 (0.89–1.00); and NONE sensitivity 0.91 (0.88–0.94) and specificity 1.00 (0.70–1.00).

**Table 2:** Bivariate diagnostic test-accuracy summary after de-duplication.

Strategy	Datasets	Arm-level observations	Sensitivity (95% CI)	Specificity (95% CI)	Random-effect SDs (logit Se / Sp)
Overall	46	4901	0.94 (0.92-0.95)	1.00 (0.98-1.00)	0.88 / 3.87
ROSE	17	1655	0.96 (0.94-0.97)	1.00 (0.94-1.00)	1.00 / 2.31
MOSE	10	936	0.94 (0.91-0.96)	1.00 (0.89-1.00)	0.74 / 3.58
NONE	19	2310	0.91 (0.88-0.94)	1.00 (0.70-1.00)	0.74 / 3.13

Se, sensitivity; Sp, specificity. Estimates come from the bivariate binomial-normal random-effects model used in the revised manuscript. Conventional univariate I2 values, crude strategy pooling, and an unreported HSROC AUC were not substituted for the bivariate summary

**Figure 3:**
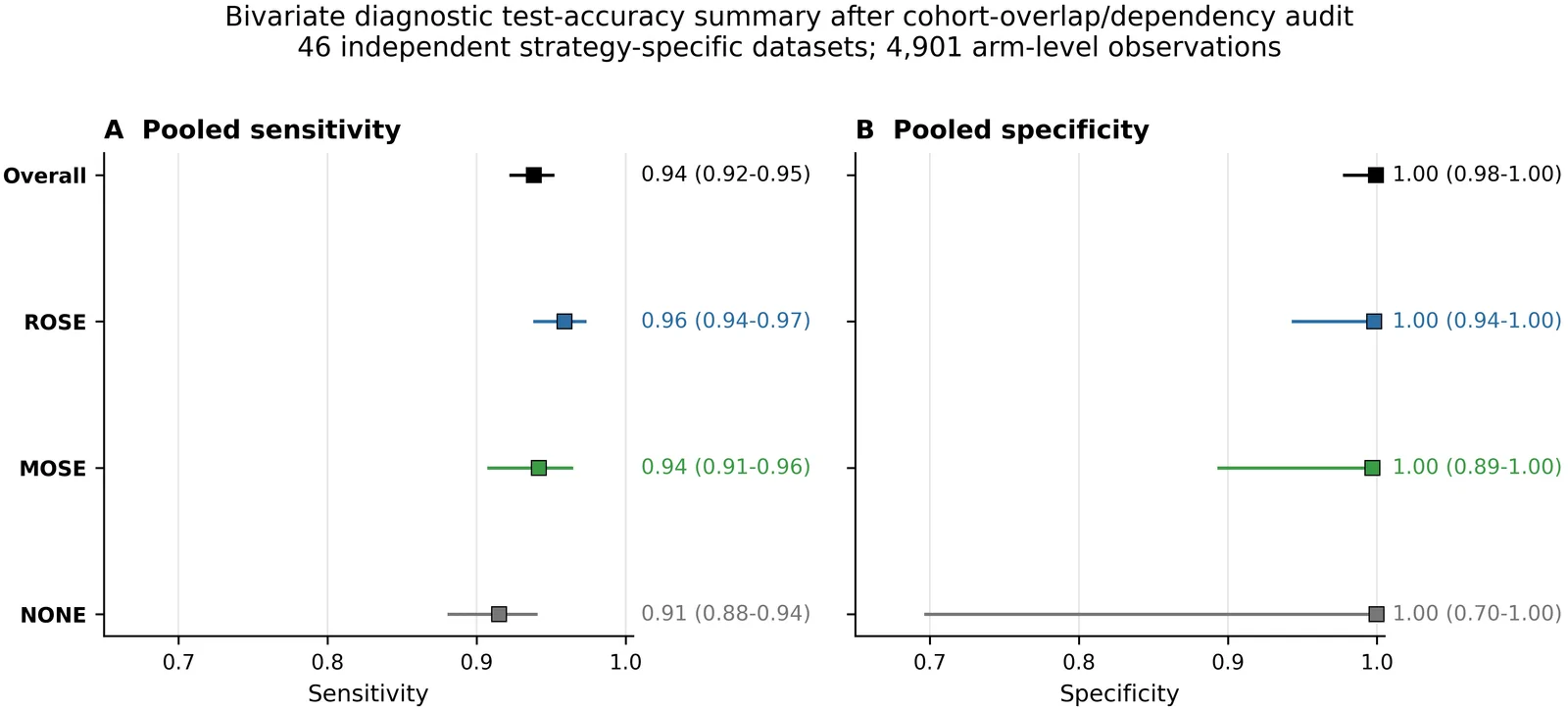
Coupled forest plot of bivariate pooled sensitivity and specificity for the primary DTA dataset and by on-site evaluation strategy. **(A)** Pooled sensitivity; **(B)** pooled specificity. Squares indicate summary estimates; horizontal lines indicate 95% confidence intervals.

**Figure 4:**
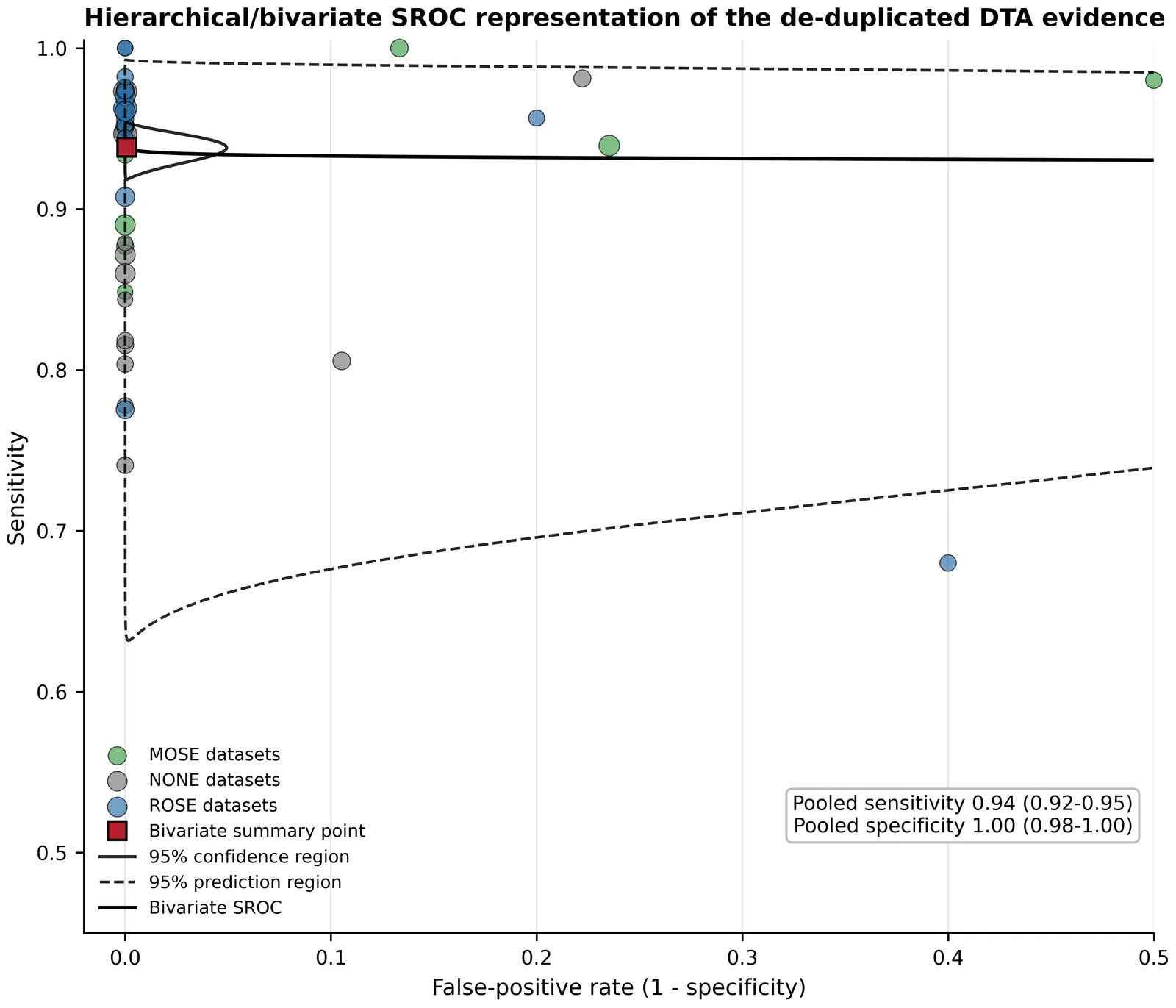
Hierarchical summary receiver operating characteristic representation of the primary DTA evidence. Colored circles represent individual diagnostic datasets, with colors denoting on-site evaluation strategy; the square is the bivariate summary point, the solid contour is the 95% confidence region, and the dashed contour is the 95% prediction region.

### 3.5 Direct comparisons of composite diagnostic accuracy

Fifteen independent direct comparisons contributed to four pooled contrasts (**Figure 5**, **Table 3**, **Supplementary Table S6**). In FNA studies, ROSE was associated with higher odds of a correct diagnosis than NONE (OR 5.56, 95% CI 1.76–17.62; k = 4; I² = 0%). In FNB studies, the corresponding estimate was close to the null and imprecise (OR 1.08, 95% CI 0.42–2.79; k = 4; I² = 0%). The exploratory interaction between the FNA and FNB strata was P = 0.012. MOSE versus NONE in FNB yielded OR 1.31 (0.60–2.87; k = 4), whereas MOSE versus ROSE was highly uncertain (OR 1.53, 0.05–49.13; k = 3; I² = 57%). The latter interval is too wide to support either equivalence or meaningful superiority. Randomized-comparison-only estimates are displayed in **Supplementary Figure S2**, and explicitly standardized-MOSE analyses are displayed in **Supplementary Figure S3**.

**Figure 5:**
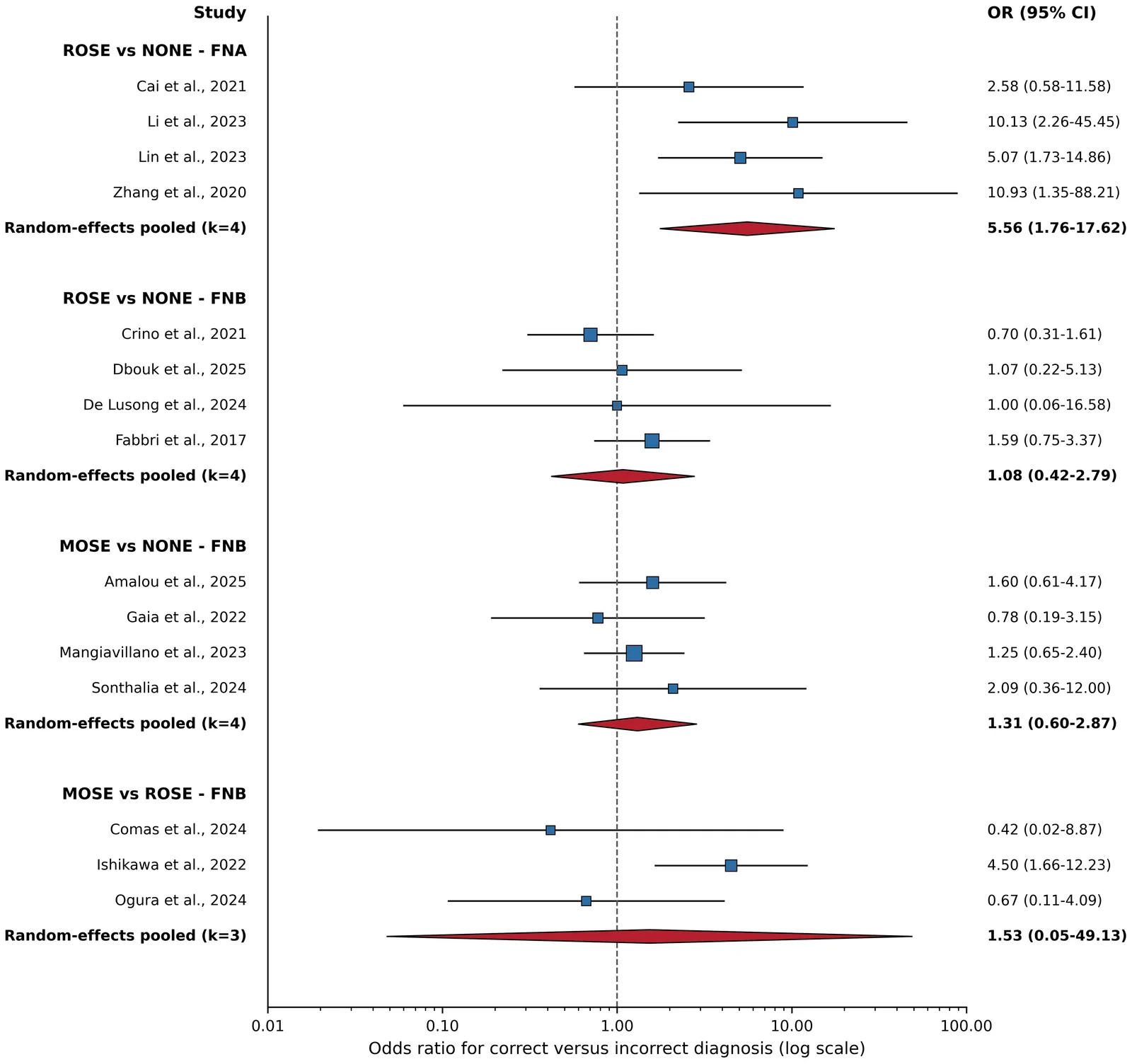
Classic forest plot of independent direct comparisons for correct versus incorrect diagnosis. Study squares and 95% confidence intervals are shown on a logarithmic odds-ratio scale; pooled random-effects estimates are shown as diamonds. ROSE versus NONE is stratified by FNA and FNB.

**Table 3:** Independent direct comparisons and pooled absolute sensitivity/specificity differences.

Comparison	Metric	k	Estimate	95% CI	I2 (%)	tau2	P
ROSE vs NONE - FNA	Composite OR	4	5.56	1.76-17.62	0.0	0.000	0.018
ROSE vs NONE - FNB	Composite OR	4	1.08	0.42-2.79	0.0	0.052	0.812
MOSE vs NONE - FNB	Composite OR	4	1.31	0.60-2.87	0.0	0.000	0.355
MOSE vs ROSE - FNB	Composite OR	3	1.53	0.05-49.13	57.3	1.087	0.648
ROSE vs NONE - FNA	Sensitivity difference	4	+0.164	+0.071 to +0.257	0.0	0.000	0.011
ROSE vs NONE - FNA	Specificity difference	4	+0.000	-0.000 to +0.000	13.9	0.000	1.000
ROSE vs NONE - FNB	Sensitivity difference	4	-0.004	-0.040 to +0.031	0.0	0.000	0.730
ROSE vs NONE - FNB	Specificity difference	4	+0.000	-0.000 to +0.000	0.0	0.000	1.000
MOSE vs NONE - FNB	Sensitivity difference	4	+0.039	-0.052 to +0.129	0.0	0.000	0.270
MOSE vs NONE - FNB	Specificity difference	4	+0.000	-0.000 to +0.000	0.0	0.000	1.000
MOSE vs ROSE - FNB	Sensitivity difference	3	+0.086	-0.342 to +0.514	89.6	0.026	0.478
MOSE vs ROSE - FNB	Specificity difference	3	-0.037	-0.266 to +0.191	20.4	0.004	0.554

Composite OR >1 favors the first-listed strategy for correct versus incorrect diagnosis. Absolute differences are first strategy minus comparator. The ROSE-versus-NONE FNA/FNB interaction is exploratory (P=0.012).

### 3.6 Sensitivity and specificity differences

The sensitivity analysis clarified what the composite endpoint was capturing (**Figures 6A, B**, **Table 3**). For ROSE versus NONE in FNA, the pooled absolute sensitivity difference was +0.164 (95% CI + 0.071 to +0.257; P = 0.011), while no measurable pooled specificity difference was detected at the reported precision. In FNB, ROSE versus NONE showed a sensitivity difference of −0.004 (−0.040 to +0.031; P = 0.730) and again no measurable specificity difference. MOSE versus NONE in FNB showed a sensitivity difference of +0.039 (−0.052 to +0.129), and MOSE versus ROSE showed +0.086 (−0.342 to +0.514). Study-level component differences are shown in **Supplementary Figure S4**. These results support interpretation of the FNA-era composite benefit as primarily sensitivity-driven rather than as a broad improvement in both dimensions of diagnostic performance.

**Figure 6:**
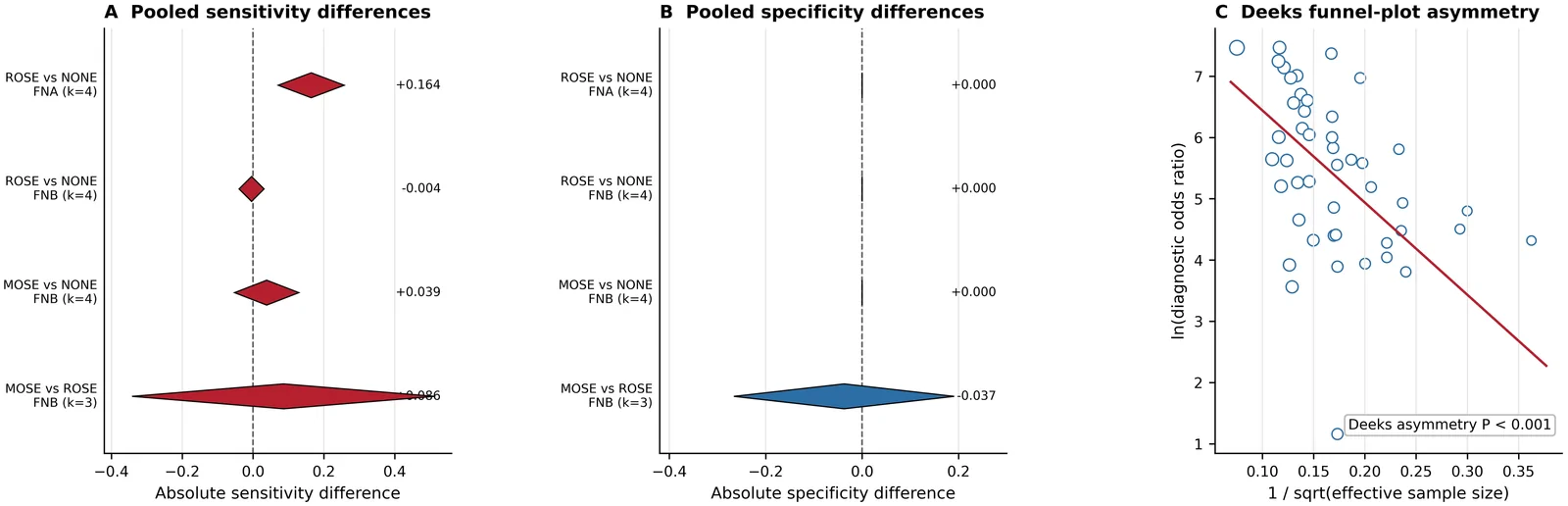
Component diagnostic outcomes and small-study effects. **(A)** Random-effects pooled absolute differences in sensitivity. **(B)** Corresponding absolute differences in specificity. **(C)** Deeks-style regression asymmetry plot based on the 46-dataset primary DTA analysis.

### 3.7 Small-study effects and certainty

Deeks’ regression showed strong asymmetry (P <0.001; **Figure 6C**), consistent with small-study effects or other size-related bias in the diagnostic evidence base. Because asymmetry can also reflect heterogeneity in design and thresholds, it was treated as a warning signal rather than proof of publication bias. In combination with methodological heterogeneity and sparse comparative evidence, this finding contributed to downgrading the overall DTA certainty to low and several comparative/NMA estimates to very low (**Supplementary Table S4**, **Supplementary Figure S5**).

### 3.8 Exploratory network meta-analysis

The network contained ROSE, MOSE, and NONE and was informed by the independent direct comparisons shown in **Figure 7**. No network comparison excluded the null: ROSE versus NONE OR 1.67 (95% CI 0.94–2.95), MOSE versus NONE OR 1.89 (0.86–4.20), and MOSE versus ROSE OR 1.14 (0.48–2.71) (**Figure 8**, **Table 4**). The local loop inconsistency factor was 0.69 (SE 0.91; 95% CI −1.09 to 2.47; P = 0.449). Thus, no statistically detectable local inconsistency was identified, although this test has limited power in a three-node network with few direct comparisons.

**Figure 7:**
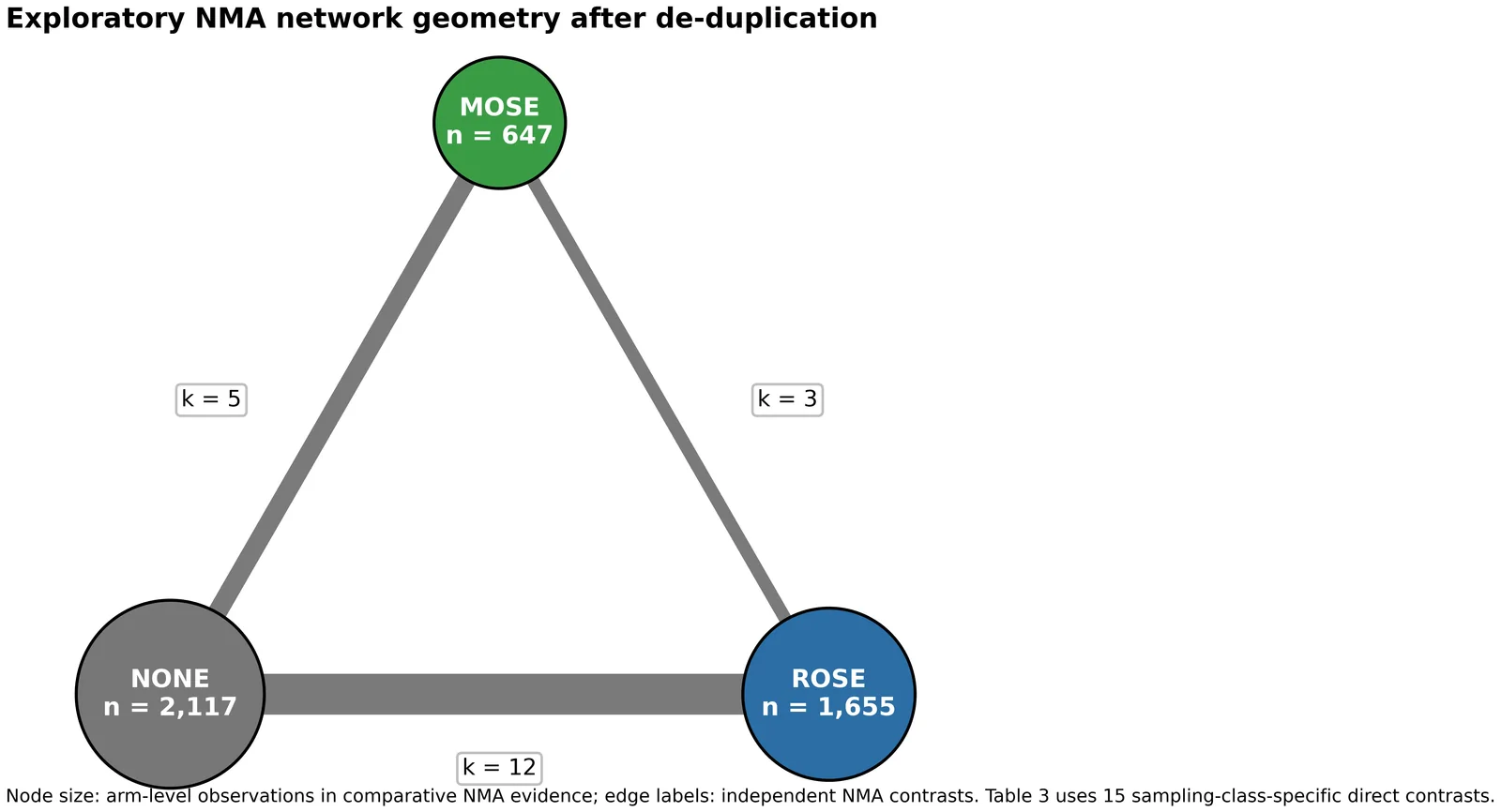
Network geometry for the exploratory three-node NMA. Line width reflects the number of independent direct comparisons; node size reflects the amount of comparative evidence contributing to each strategy.

**Figure 8:**
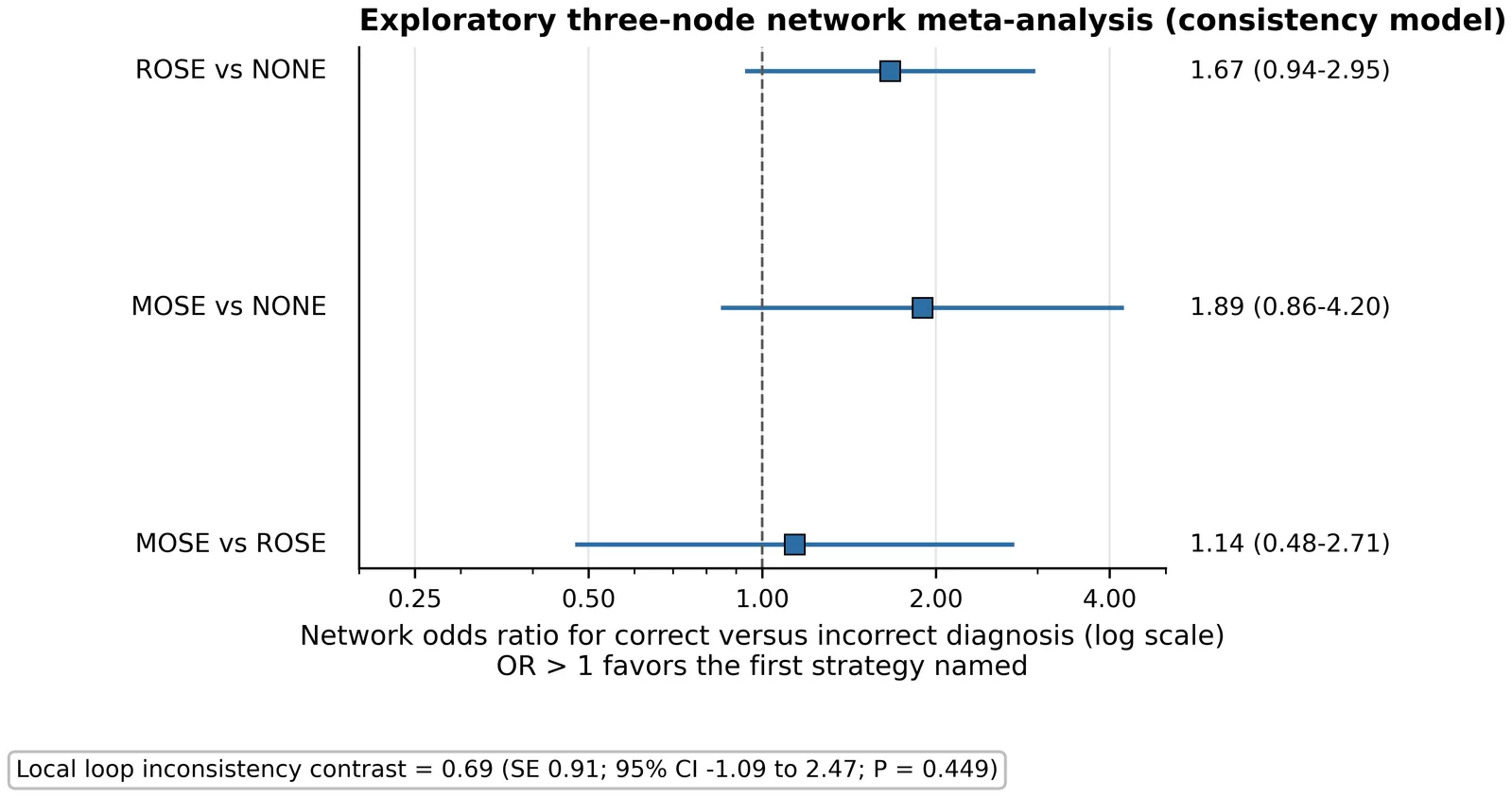
Exploratory NMA forest plot for correct versus incorrect diagnosis. Odds ratios greater than 1 favor the first-listed strategy. None of the three network 95% confidence intervals excluded 1.

**Table 4:** Exploratory network meta-analysis after de-duplication.

Network comparison / statistic	Estimate	95% CI	Interpretation	Certainty
ROSE vs NONE	1.67	0.94-2.95	95% CI includes 1	Very low
MOSE vs NONE	1.89	0.86-4.20	95% CI includes 1	Very low
MOSE vs ROSE	1.14	0.48-2.71	95% CI includes 1	Very low
Local loop inconsistency contrast	0.69	-1.09 to 2.47	P=0.449	Not a certainty rating

The NMA combines all independent study-level contrasts available for the exploratory network, including mixed/unspecified sampling workflows. **Figure 7** therefore contains 12 ROSE-NONE, 5 MOSE-NONE, and 3 MOSE-ROSE network edges, whereas the sampling-class-specific direct meta-analyses in **Table 3** use 15 direct contrasts. No network treatment comparison excluded the null.

## 4 Discussion

This review assessed two questions: how accurately EUS-guided tissue acquisition diagnoses solid pancreatic masses, and whether one on-site evaluation strategy improves diagnostic accuracy over another within a comparable sampling platform. Overall EUS-TA performance was high, but the direct comparative results were more restrained. ROSE was associated with a clear benefit over NONE in FNA studies, whereas the corresponding FNB estimate was close to the null and imprecise. MOSE did not show a statistically clear advantage over NONE in FNB studies, and the direct MOSE-versus-ROSE evidence was too sparse and heterogeneous to establish equivalence. This pattern is consistent with the broader literature showing that the incremental value of on-site evaluation depends on needle design, sampling workflow, and the availability and role of cytopathology (6–8, 11, 12, 18).

The contrast between FNA and FNB is biologically and technically plausible. FNA depends primarily on cytologic material, so immediate adequacy assessment can help determine whether additional passes are needed (3, 11, 16). By contrast, contemporary FNB needles, particularly end-cutting designs, are intended to obtain larger architecturally preserved tissue cores and may reduce the incremental information provided by immediate cytology (4, 6–8, 17). The approximately 16-percentage-point sensitivity advantage observed for ROSE in FNA studies, together with the near-null sensitivity difference in FNB studies, is consistent with this platform-dependent interpretation and with prior evidence that the benefit of ROSE diminishes when newer end-cutting FNB needles are used (6, 18). Nevertheless, the FNA-versus-FNB interaction remains exploratory because relatively few studies contributed to each stratum and studies were not randomized across needle technologies.

Our findings also place the prior FNB-specific meta-analysis in context. Facciorusso and colleagues reported higher diagnostic accuracy for FNB plus ROSE overall, but no significant improvement when newer end-cutting needles were analyzed separately (18). An RCT-only meta-analysis of ROSE versus no-ROSE and a recent meta-analysis of FNB pass number similarly indicate that procedural performance is strongly conditioned by needle generation and sampling protocol (12, 17). Our FNB-specific estimate (OR 1.08, 95% CI 0.42–2.79) is compatible with this technology-dependent interpretation. Differences across reviews may reflect needle generation, study eligibility, outcome definition, and the composition of the evidence base.

MOSE remains attractive because it may reduce dependence on cytopathology staffing while preserving immediate feedback about gross specimen adequacy. Prospective and randomized studies have evaluated visible-core length, macroscopic scoring, stereomicroscopic assessment, and direct MOSE comparisons with either ROSE or fixed-pass/no-on-site workflows (13–15, 34, 40, 42, 47, 48, 51–53). However, the current evidence does not justify treating MOSE and ROSE as interchangeable. MOSE versus NONE was compatible with both benefit and no effect, and the direct MOSE-versus-ROSE interval extended from a large disadvantage to a very large advantage. Protocols also differed in what constituted an adequate macroscopic core, whether stereomicroscopy or formal scoring was used, how operators were trained, and whether the same needle type was used in both study arms (13–15, 40, 42, 48). These sources of heterogeneity undermine strong rank-ordering of on-site strategies.

The distinction between composite diagnostic accuracy and the component measures is also important. Diagnostic-test meta-analysis is generally more informative when sensitivity and specificity are modeled jointly rather than collapsed into a single accuracy measure (22, 23). An OR based on correct versus incorrect diagnosis can be dominated by sensitivity when specificity is close to 100%, as occurred in many pancreatic-mass studies. A composite endpoint can therefore appear favorable without demonstrating improvement across both false-negative and false-positive errors. The added absolute-difference analyses show that the FNA-era ROSE signal was predominantly sensitivity-driven. Conversely, the near-zero specificity differences should not be overinterpreted as proof of exact equality; false positives were sparse, and several specificity intervals were intrinsically unstable.

Several limitations should be considered. First, eligible studies span FNA, FNB, and mixed workflows, multiple needle designs, variable operator experience, nonuniform ROSE and MOSE definitions, and differing NONE workflows; these are recognized sources of variation in EUS-guided pancreatic tissue acquisition (7, 8, 11, 13–18). Second, the exact recruitment relationship between some abstracts and full reports cannot always be established from published information; when dependency was plausible, the more complete report was preferentially retained to reduce unit-of-analysis bias. Third, positive Deeks’ asymmetry and heterogeneous QUADAS-2 judgments reduce confidence in the apparent high diagnostic performance (21, 24). Risk-of-bias concerns in patient selection, blinding, or reference-standard verification may influence pooled comparative estimates, particularly when larger or more precise studies carry substantial weight. Fourth, the NMA is exploratory because the network is sparse and transitivity across needle generations and on-site protocols cannot be assumed. Fifth, the registered protocol initially emphasized FNB, whereas the final eligibility framework encompassed EUS-TA including FNA and mixed workflows; this post-registration broadening introduces potential analytical flexibility. Finally, several studies remained at high or unclear risk of bias, and residual methodological heterogeneity cannot be eliminated by statistical modeling.

For practice, the most supportable conclusion is conditional rather than universal. ROSE may still be useful where FNA remains common, where prior sampling is nondiagnostic, or where immediate cytologic decisions change the procedure (7, 11, 12, 16, 18, 56). With modern FNB, routine ROSE should not be assumed to improve diagnostic accuracy, and operational factors such as pathologist availability, procedure time, tissue requirements for ancillary testing, and local expertise may dominate the decision (6–10, 12, 17, 18, 36, 37). MOSE may be reasonable as a pragmatic specimen-feedback strategy, and some randomized studies suggest that it can reduce the number of passes, but current comparative data are too imprecise to establish that it replaces ROSE in all settings (13–15, 34, 40, 47, 48, 51, 52). Operational adaptations such as trained-endosonographer ROSE/ROLE and stereomicroscopic MOSE further suggest that implementation may depend on local expertise and workflow (33, 42, 55). Future trials should predefine needle type and MOSE threshold, report 2 × 2 data and molecular-tissue adequacy separately, preserve paired covariance when the same patient undergoes multiple assessments, and prospectively register how repeated or overlapping cohorts will be handled.

## 5 Conclusions

EUS-guided tissue acquisition demonstrated high overall diagnostic performance for solid pancreatic masses. ROSE was associated with higher diagnostic accuracy in FNA-based workflows, driven primarily by improved sensitivity, whereas no clear incremental benefit was detected in FNB studies. MOSE did not show a statistically clear advantage over NONE, and evidence comparing MOSE with ROSE remained imprecise. The exploratory NMA likewise found no statistically clear superiority among ROSE, MOSE, and NONE. These findings support a platform-dependent interpretation of on-site evaluation, with clinical decisions guided by needle technology, local cytopathology resources, and tissue requirements for ancillary testing.

## Data Availability

The datasets generated and/or analyzed during the current study are available from the corresponding author on reasonable request. Requests to access these datasets should be directed to: BC, 583186126@qq.com.

## Supplementary material

Supplementary Figure 1 Full 46-dataset coupled forest plot of sensitivity and specificity for the primary DTA. Study-level exact binomial confidence intervals are shown; the bivariate pooled estimates are displayed at the bottom.

Supplementary Figure 2 Randomized-comparison-only direct meta-analysis for correct versus incorrect diagnosis. Panel A shows ROSE versus NONE in FNB studies; Panel B shows MOSE versus NONE in FNB studies; Panel C shows MOSE versus ROSE in FNB studies. A single eligible randomized comparison is shown without pooling when meta-analysis is not informative.

Supplementary Figure 3 Restricted analysis of explicitly standardized MOSE definitions. Panel A shows standardized MOSE versus NONE in FNB studies; Panel B shows standardized MOSE versus ROSE in FNB studies. Standardized MOSE indicates that an explicit score or core-length threshold was confirmed in the available report.

Supplementary Figure 4 Study-level component diagnostic outcomes. Random-effects pooled absolute differences in sensitivity and specificity are shown for the four independent direct-comparison contrasts.

Supplementary Figure 5 Certainty-of-evidence profile. The figure summarizes certainty judgments for overall DTA, direct comparative estimates, and the exploratory network meta-analysis after considering risk of bias, inconsistency, indirectness, imprecision, and small-study effects.

Supplementary Table 1 PRISMA 2020 checklist.

Supplementary Table 2 Electronic database search strategies.

Supplementary Table 3 Diagnostic datasets included in the primary DTA.

Supplementary Table 4 Certainty-of-evidence assessment.

Supplementary Table 5 Cohort-overlap and data-dependency assessment of all 67 extracted datasets.

Supplementary Table 6 Study-level independent direct comparisons.
