# Supplementary material for: Macroscopic versus rapid on-site evaluation for EUS-guided tissue acquisition of solid pancreatic masses: a systematic review, diagnostic test accuracy meta-analysis, and network meta-analysis
Source: Front Oncol. 2026 Oct 19;16:1943706. doi: 10.3389/fonc.2026.1943706 (PMC13724458; doi:10.3389/fonc.2026.1943706)
Supplement: Supplementary file 1 [file DataSheet1.docx]

Supplementary Tables

Supplementary Table S1. PRISMA 2020 checklist.

| Section/item | Checklist content | Location in manuscript |
| --- | --- | --- |
| Title | Identify the report as a systematic review / diagnostic test-accuracy review | Title |
| Abstract | Structured summary of rationale, methods, results, and conclusions | Abstract |
| Introduction | Clinical rationale and study objectives | Introduction |
| Protocol and registration | Registration information and protocol amendment disclosure | Methods 2.1 |
| Eligibility criteria | Eligibility criteria for participants, tests, and study designs | Methods 2.2 |
| Information sources | Databases, supplementary sources, and search date | Methods 2.3 |
| Search strategy | Full database-specific search strategies | Methods 2.3; Supplementary Table S2 |
| Selection process | Screening and eligibility assessment procedures | Methods 2.3; Figure 1 |
| Data collection process | Data extraction and management | Methods 2.4 |
| Risk of bias assessment | QUADAS-2 domains and approach | Methods 2.5; Figure 2 |
| Effect measures | Sensitivity, specificity, odds ratio, absolute differences | Methods 2.6–2.8 |
| Synthesis methods | Bivariate DTA, direct comparative meta-analysis, exploratory NMA | Methods 2.6–2.9 |
| Reporting-bias assessment | Deeks asymmetry test | Methods 2.9; Figure 6C |
| Study selection results | Flow of records and included studies | Results 3.1; Figure 1 |
| Study characteristics | Characteristics of retained diagnostic datasets | Table 1; Supplementary Table S3 |
| Risk of bias in studies | Summary of QUADAS-2 judgments | Results 3.2; Figure 2 |
| Results of individual studies | Study-level diagnostic data and direct comparisons | Supplementary Tables S3, S5, and S6 |
| Results of syntheses | Pooled DTA, direct comparisons, component outcomes, and NMA | Results 3.3–3.8; Tables 2–4; Figures 3–8 |
| Certainty assessment | Certainty ratings and downgrade reasons | Results 3.7; Supplementary Table S4; Supplementary Figure S5 |
| Discussion | Interpretation, limitations, and implications | Discussion |
| Availability of data | Availability of data and code | Declarations |

Supplementary Table S2. Electronic database search strategies.

| Database | Last search date | Search strategy |
| --- | --- | --- |
| PubMed | 15 Dec 2025 | (pancrea*[tiab] OR pancreatic neoplasm*[tiab] OR pancreatic mass*[tiab] OR pancreatic lesion*[tiab]) AND ((endoscopic ultrasound[tiab] OR EUS[tiab]) AND (fine needle aspiration[tiab] OR FNA[tiab] OR fine needle biopsy[tiab] OR FNB[tiab] OR tissue acquisition[tiab])) AND ((rapid on-site evaluation[tiab] OR ROSE[tiab]) OR (macroscopic on-site evaluation[tiab] OR MOSE[tiab] OR visible core[tiab]) OR (without on-site evaluation[tiab] OR no on-site evaluation[tiab])) |
| Embase | 15 Dec 2025 | ('pancreas tumor'/exp OR pancrea*:ti,ab OR 'pancreatic mass':ti,ab OR 'pancreatic lesion':ti,ab) AND (('endoscopic ultrasonography'/exp OR eus:ti,ab) AND ('fine needle aspiration'/exp OR 'fine needle biopsy'/exp OR fna:ti,ab OR fnb:ti,ab OR 'tissue acquisition':ti,ab)) AND (('rapid on-site evaluation':ti,ab OR rose:ti,ab) OR ('macroscopic on-site evaluation':ti,ab OR mose:ti,ab OR 'visible core':ti,ab) OR ('without on-site evaluation':ti,ab OR 'no on-site evaluation':ti,ab)) |
| CENTRAL | 15 Dec 2025 | (pancreatic OR pancreas) near/3 (mass OR lesion OR neoplasm OR tumour OR tumor) AND (EUS OR endoscopic ultrasound) AND ((fine needle aspiration OR FNA) OR (fine needle biopsy OR FNB) OR tissue acquisition) AND (ROSE OR rapid on-site evaluation OR MOSE OR macroscopic on-site evaluation OR no on-site evaluation) |
| WanFang | 15 Dec 2025 | 主题:(胰腺 OR 胰腺肿块 OR 胰腺占位) AND 主题:(超声内镜 OR EUS) AND 主题:(细针穿刺 OR FNA OR 细针活检 OR FNB OR 组织获取) AND 主题:(快速现场评价 OR ROSE OR 宏观现场评价 OR MOSE OR 无现场评价) |

Supplementary Table S3. Diagnostic datasets included in the primary DTA.

| Study ID | Arm | Author | Year | Strategy | Sampling method | Analyzable n | TP | FP | FN | TN | Audit decision |
| --- | --- | --- | --- | --- | --- | --- | --- | --- | --- | --- | --- |
| Amalou2025 | 1 | Amalou | 2025 | MOSE | FNB | 74 | 57 | 0 | 8 | 9 | Retained in primary overall DTA after cohort/dependency audit. |
| Amalou2025 | 2 | Amalou | 2025 | NONE | FNB | 74 | 53 | 0 | 12 | 9 | Retained in primary overall DTA after cohort/dependency audit. |
| Cai2021 | 1 | Cai | 2021 | ROSE | FNA | 51 | 44 | 1 | 2 | 4 | Retained in primary overall DTA after cohort/dependency audit. |
| Cai2021 | 2 | Cai | 2021 | NONE | FNA | 36 | 27 | 0 | 5 | 4 | Retained in primary overall DTA after cohort/dependency audit. |
| Cai2024 | 1 | Cai | 2024 | ROSE | EUS-TA / not fully specified | 75 | 65 | 0 | 3 | 7 | Retained in primary overall DTA after cohort/dependency audit. |
| Cai2024 | 2 | Cai | 2024 | NONE | EUS-TA / not fully specified | 62 | 45 | 0 | 11 | 6 | Retained in primary overall DTA after cohort/dependency audit. |
| Chong, C. C. N.2020 | 1 | Chong, C. C. N. | 2020 | MOSE | EUS-TA / not fully specified | 61 | 40 | 0 | 1 | 20 | Retained in primary overall DTA after cohort/dependency audit. |
| Chong, C. C. N.2020 | 2 | Chong, C. C. N. | 2020 | NONE | EUS-TA / not fully specified | 80 | 52 | 6 | 1 | 21 | Retained in primary overall DTA after cohort/dependency audit. |
| Comas2024 | 1 | Comas | 2024 | MOSE | FNB | 90 | 75 | 2 | 0 | 13 | Retained in primary overall DTA after cohort/dependency audit. |
| Comas2024 | 2 | Comas | 2024 | ROSE | FNB | 42 | 35 | 0 | 0 | 7 | Retained in primary overall DTA after cohort/dependency audit. |
| Crinò2021 | 1 | Crinò | 2021 | ROSE | FNB | 385 | 359 | 0 | 14 | 12 | Retained in primary overall DTA after cohort/dependency audit. |
| Crinò2021 | 2 | Crinò | 2021 | NONE | FNB | 386 | 361 | 0 | 10 | 15 | Retained in primary overall DTA after cohort/dependency audit. |
| Dbouk2025 | 1 | Dbouk | 2025 | ROSE | FNB | 44 | 38 | 0 | 2 | 4 | Retained in primary overall DTA after cohort/dependency audit. |
| Dbouk2025 | 2 | Dbouk | 2025 | NONE | FNB | 186 | 164 | 0 | 9 | 13 | Retained in primary overall DTA after cohort/dependency audit. |
| De Lusong2024 | 1 | De Lusong | 2024 | ROSE | FNB | 39 | 37 | 0 | 1 | 1 | Retained in primary overall DTA after cohort/dependency audit. |
| De Lusong2024 | 2 | De Lusong | 2024 | NONE | FNB | 39 | 36 | 0 | 1 | 2 | Retained in primary overall DTA after cohort/dependency audit. |
| Fabbri2017 | 1 | Fabbri | 2017 | ROSE | FNB | 140 | 108 | 0 | 11 | 21 | Retained in primary overall DTA after cohort/dependency audit. |
| Fabbri2017 | 2 | Fabbri | 2017 | NONE | FNB | 193 | 156 | 0 | 23 | 14 | Retained in primary overall DTA after cohort/dependency audit. |
| Gaia2022 | 1 | Gaia | 2022 | MOSE | FNB | 38 | 28 | 0 | 5 | 5 | Retained in primary overall DTA after cohort/dependency audit. |
| Gaia2022 | 2 | Gaia | 2022 | NONE | FNB | 38 | 29 | 0 | 4 | 5 | Retained in primary overall DTA after cohort/dependency audit. |
| Iglesias-Garcia2024a | 1 | Iglesias-Garcia | 2024 | ROSE | Mixed/unclear FNA-FNB | 179 | 162 | 0 | 5 | 12 | Retained in primary overall DTA after cohort/dependency audit. |
| Iglesias-Garcia2024a | 2 | Iglesias-Garcia | 2024 | NONE | Mixed/unclear FNA-FNB | 387 | 319 | 0 | 18 | 50 | Retained in primary overall DTA after cohort/dependency audit. |
| Ishikawa2022 | 1 | Ishikawa | 2022 | MOSE | FNB | 60 | 49 | 5 | 1 | 5 | Retained in primary overall DTA after cohort/dependency audit. |
| Ishikawa2022 | 2 | Ishikawa | 2022 | ROSE | FNB | 60 | 34 | 4 | 16 | 6 | Retained in primary overall DTA after cohort/dependency audit. |
| Li2023a | 1 | Li | 2023 | ROSE | FNA | 52 | 40 | 0 | 2 | 10 | Retained in primary overall DTA after cohort/dependency audit. |
| Li2023b | 2 | Li | 2023 | ROSE | FNA | 45 | 34 | 0 | 0 | 11 | Retained in primary overall DTA after cohort/dependency audit. |
| Li2023c | 3 | Li | 2023 | NONE | FNA | 91 | 58 | 2 | 14 | 17 | Retained in primary overall DTA after cohort/dependency audit. |
| Lin2023a | 1 | Lin | 2023 | ROSE | FNA | 59 | 47 | 0 | 3 | 9 | Retained in primary overall DTA after cohort/dependency audit. |
| Lin2023b | 2 | Lin | 2023 | ROSE | FNA | 42 | 34 | 0 | 2 | 6 | Retained in primary overall DTA after cohort/dependency audit. |
| Lin2023c | 3 | Lin | 2023 | NONE | FNA | 67 | 40 | 0 | 14 | 13 | Retained in primary overall DTA after cohort/dependency audit. |
| Mangiavillano, B.2023 | 1 | Mangiavillano, B. | 2023 | MOSE | FNB | 190 | 154 | 0 | 19 | 17 | Retained in primary overall DTA after cohort/dependency audit. |
| Mangiavillano, B.2023 | 2 | Mangiavillano, B. | 2023 | NONE | FNB | 180 | 135 | 0 | 22 | 23 | Retained in primary overall DTA after cohort/dependency audit. |
| Naidu2021a | 1 | Naidu | 2021 | ROSE | Mixed/unclear FNA-FNB | 104 | 69 | 0 | 20 | 15 | Retained in primary overall DTA after cohort/dependency audit. |
| Naidu2021a | 2 | Naidu | 2021 | NONE | Mixed/unclear FNA-FNB | 106 | 99 | 0 | 5 | 2 | Retained in primary overall DTA after cohort/dependency audit. |
| Ogura2024 | 1 | Ogura | 2024 | ROSE | FNB | 85 | 74 | 0 | 2 | 9 | Retained in primary overall DTA after cohort/dependency audit. |
| Ogura2024 | 2 | Ogura | 2024 | MOSE | FNB | 86 | 74 | 0 | 3 | 9 | Retained in primary overall DTA after cohort/dependency audit. |
| Okasha2024a | 1 | Okasha | 2024 | MOSE | Mixed/unclear FNA-FNB | 215 | 186 | 4 | 12 | 13 | Retained in primary overall DTA after cohort/dependency audit. |
| Ratnaraj2018 | 1 | Ratnaraj | 2018 | ROSE | Mixed/unclear FNA-FNB | 193 | 171 | 0 | 7 | 15 | Retained in primary overall DTA after cohort/dependency audit. |
| Ratnaraj2018 | 2 | Ratnaraj | 2018 | NONE | Mixed/unclear FNA-FNB | 80 | 71 | 0 | 3 | 6 | Retained in primary overall DTA after cohort/dependency audit. |
| So2021 | 1 | So | 2021 | MOSE | FNB | 74 | 29 | 0 | 1 | 44 | Retained in primary overall DTA after cohort/dependency audit. |
| Sonthalia, N.2024 | 1 | Sonthalia, N. | 2024 | MOSE | FNB | 48 | 28 | 0 | 2 | 18 | Retained in primary overall DTA after cohort/dependency audit. |
| Sonthalia, N.2024 | 2 | Sonthalia, N. | 2024 | NONE | FNB | 48 | 14 | 0 | 4 | 30 | Retained in primary overall DTA after cohort/dependency audit. |
| Xu2024a | 1 | Xu | 2024 | NONE | FNB | 96 | 73 | 0 | 2 | 21 | Retained in primary overall DTA after cohort/dependency audit. |
| Xu2024b | 2 | Xu | 2024 | NONE | FNB | 97 | 71 | 0 | 2 | 24 | Retained in primary overall DTA after cohort/dependency audit. |
| Zhang2020 | 1 | Zhang | 2020 | ROSE | FNA | 60 | 55 | 0 | 1 | 4 | Retained in primary overall DTA after cohort/dependency audit. |
| Zhang2020 | 2 | Zhang | 2020 | NONE | FNA | 64 | 45 | 0 | 10 | 9 | Retained in primary overall DTA after cohort/dependency audit. |

Supplementary Table S4. Certainty-of-evidence assessment.

| Evidence question | Evidence base | Certainty | Main downgrade reasons |
| --- | --- | --- | --- |
| Overall DTA | 46 datasets | Low | Risk-of-bias concerns; substantial methodological heterogeneity; positive Deeks asymmetry. |
| ROSE vs NONE, FNA | 4 direct comparisons | Low | Small evidence base; imprecision despite consistent direction; platform-era indirectness. |
| ROSE vs NONE, FNB | 4 direct comparisons | Low | Imprecision and heterogeneity in FNB workflow; confidence interval includes clinically relevant effects. |
| MOSE vs NONE, FNB | 4 direct comparisons | Very low | Sparse studies; nonuniform MOSE definitions; imprecision. |
| MOSE vs ROSE, FNB | 3 direct comparisons | Very low | Very serious imprecision; heterogeneity; extremely wide confidence interval. |
| Exploratory NMA | Sparse 3-node network | Very low | Transitivity concerns, sparse network, composite endpoint, and small-study effects. |

Supplementary Table S5. Cohort-overlap and data-dependency assessment of all 67 extracted datasets

| Study ID | Arm | Author | Year | Strategy | Sampling | n | Audit cluster | Audit decision | Primary DTA |
| --- | --- | --- | --- | --- | --- | --- | --- | --- | --- |
| Amalou2025 | 1 | Amalou | 2025 | MOSE | FNB | 74 |  | Retained in primary overall DTA after cohort/dependency audit. | 1 |
| Amalou2025 | 2 | Amalou | 2025 | NONE | FNB | 74 |  | Retained in primary overall DTA after cohort/dependency audit. | 1 |
| Cai2021 | 1 | Cai | 2021 | ROSE | FNA | 51 |  | Retained in primary overall DTA after cohort/dependency audit. | 1 |
| Cai2021 | 2 | Cai | 2021 | NONE | FNA | 36 |  | Retained in primary overall DTA after cohort/dependency audit. | 1 |
| Cai2024 | 1 | Cai | 2024 | ROSE | EUS-TA / not fully specified | 75 |  | Retained in primary overall DTA after cohort/dependency audit. | 1 |
| Cai2024 | 2 | Cai | 2024 | NONE | EUS-TA / not fully specified | 62 |  | Retained in primary overall DTA after cohort/dependency audit. | 1 |
| Chong, C. C. N.2020 | 1 | Chong, C. C. N. | 2020 | MOSE | EUS-TA / not fully specified | 61 |  | Retained in primary overall DTA after cohort/dependency audit. | 1 |
| Chong, C. C. N.2020 | 2 | Chong, C. C. N. | 2020 | NONE | EUS-TA / not fully specified | 80 |  | Retained in primary overall DTA after cohort/dependency audit. | 1 |
| Comas2024 | 1 | Comas | 2024 | MOSE | FNB | 90 |  | Retained in primary overall DTA after cohort/dependency audit. | 1 |
| Comas2024 | 2 | Comas | 2024 | ROSE | FNB | 42 |  | Retained in primary overall DTA after cohort/dependency audit. | 1 |
| Crinò2021 | 1 | Crinò | 2021 | ROSE | FNB | 385 |  | Retained in primary overall DTA after cohort/dependency audit. | 1 |
| Crinò2021 | 2 | Crinò | 2021 | NONE | FNB | 386 |  | Retained in primary overall DTA after cohort/dependency audit. | 1 |
| Dbouk2025 | 1 | Dbouk | 2025 | ROSE | FNB | 44 |  | Retained in primary overall DTA after cohort/dependency audit. | 1 |
| Dbouk2025 | 2 | Dbouk | 2025 | NONE | FNB | 186 |  | Retained in primary overall DTA after cohort/dependency audit. | 1 |
| De Lusong2024 | 1 | De Lusong | 2024 | ROSE | FNB | 39 |  | Retained in primary overall DTA after cohort/dependency audit. | 1 |
| De Lusong2024 | 2 | De Lusong | 2024 | NONE | FNB | 39 |  | Retained in primary overall DTA after cohort/dependency audit. | 1 |
| Fabbri2017 | 1 | Fabbri | 2017 | ROSE | FNB | 140 |  | Retained in primary overall DTA after cohort/dependency audit. | 1 |
| Fabbri2017 | 2 | Fabbri | 2017 | NONE | FNB | 193 |  | Retained in primary overall DTA after cohort/dependency audit. | 1 |
| Gaia2022 | 1 | Gaia | 2022 | MOSE | FNB | 38 |  | Retained in primary overall DTA after cohort/dependency audit. | 1 |
| Gaia2022 | 2 | Gaia | 2022 | NONE | FNB | 38 |  | Retained in primary overall DTA after cohort/dependency audit. | 1 |
| Iglesias-Garcia2024a | 1 | Iglesias-Garcia | 2024 | ROSE | Mixed/unclear FNA-FNB | 179 |  | Retained in primary overall DTA after cohort/dependency audit. | 1 |
| Iglesias-Garcia2024a | 2 | Iglesias-Garcia | 2024 | NONE | Mixed/unclear FNA-FNB | 387 |  | Retained in primary overall DTA after cohort/dependency audit. | 1 |
| Iglesias-García2024b | 1 | Iglesias-García | 2024 | ROSE | Mixed/unclear FNA-FNB | 179 | Iglesias2024-a-b | Excluded: second dependent endpoint/dataset from the same prospective registry cohort represented by Iglesias-Garcia2024a. | 0 |
| Iglesias-García2024b | 2 | Iglesias-García | 2024 | NONE | Mixed/unclear FNA-FNB | 387 | Iglesias2024-a-b | Excluded: second dependent endpoint/dataset from the same prospective registry cohort represented by Iglesias-Garcia2024a. | 0 |
| Ishikawa2022 | 1 | Ishikawa | 2022 | MOSE | FNB | 60 |  | Retained in primary overall DTA after cohort/dependency audit. | 1 |
| Ishikawa2022 | 2 | Ishikawa | 2022 | ROSE | FNB | 60 |  | Retained in primary overall DTA after cohort/dependency audit. | 1 |
| Lario-Noia2022 | 1 | Lario-Noia | 2022 | ROSE | Mixed/unclear FNA-FNB | 48 | Lario2022-Iglesias2024 | Excluded conservatively: earlier report from the same prospective EUS registry/authorship group as the later Iglesias-Garcia registry report. | 0 |
| Lario-Noia2022 | 2 | Lario-Noia | 2022 | NONE | Mixed/unclear FNA-FNB | 123 | Lario2022-Iglesias2024 | Excluded conservatively: earlier report from the same prospective EUS registry/authorship group as the later Iglesias-Garcia registry report. | 0 |
| Li2023a | 1 | Li | 2023 | ROSE | FNA | 52 |  | Retained in primary overall DTA after cohort/dependency audit. | 1 |
| Li2023b | 2 | Li | 2023 | ROSE | FNA | 45 |  | Retained in primary overall DTA after cohort/dependency audit. | 1 |
| Li2023c | 3 | Li | 2023 | NONE | FNA | 91 |  | Retained in primary overall DTA after cohort/dependency audit. | 1 |
| Lin2023a | 1 | Lin | 2023 | ROSE | FNA | 59 |  | Retained in primary overall DTA after cohort/dependency audit. | 1 |
| Lin2023b | 2 | Lin | 2023 | ROSE | FNA | 42 |  | Retained in primary overall DTA after cohort/dependency audit. | 1 |
| Lin2023c | 3 | Lin | 2023 | NONE | FNA | 67 |  | Retained in primary overall DTA after cohort/dependency audit. | 1 |
| Mangiavillano2022 | 1 | Mangiavillano | 2022 | MOSE | FNB | 190 | Mangiavillano2022-2023 | Excluded: preliminary conference report duplicates the later full randomized trial; 2023 full report retained. | 0 |
| Mangiavillano2022 | 2 | Mangiavillano | 2022 | NONE | FNB | 180 | Mangiavillano2022-2023 | Excluded: preliminary conference report duplicates the later full randomized trial; 2023 full report retained. | 0 |
| Mangiavillano, B.2023 | 1 | Mangiavillano, B. | 2023 | MOSE | FNB | 190 |  | Retained in primary overall DTA after cohort/dependency audit. | 1 |
| Mangiavillano, B.2023 | 2 | Mangiavillano, B. | 2023 | NONE | FNB | 180 |  | Retained in primary overall DTA after cohort/dependency audit. | 1 |
| Naidu2021a | 1 | Naidu | 2021 | ROSE | Mixed/unclear FNA-FNB | 104 |  | Retained in primary overall DTA after cohort/dependency audit. | 1 |
| Naidu2021a | 2 | Naidu | 2021 | NONE | Mixed/unclear FNA-FNB | 106 |  | Retained in primary overall DTA after cohort/dependency audit. | 1 |
| Naidu2021b | 1 | Naidu | 2021 | ROSE | Mixed/unclear FNA-FNB | 104 | Naidu2021-a-b | Excluded: second dependent endpoint/dataset from the same randomized cohort; Naidu2021a retained. | 0 |
| Naidu2021b | 2 | Naidu | 2021 | NONE | Mixed/unclear FNA-FNB | 106 | Naidu2021-a-b | Excluded: second dependent endpoint/dataset from the same randomized cohort; Naidu2021a retained. | 0 |
| Ogura2024 | 1 | Ogura | 2024 | ROSE | FNB | 85 |  | Retained in primary overall DTA after cohort/dependency audit. | 1 |
| Ogura2024 | 2 | Ogura | 2024 | MOSE | FNB | 86 |  | Retained in primary overall DTA after cohort/dependency audit. | 1 |
| Okasha2024a | 1 | Okasha | 2024 | MOSE | Mixed/unclear FNA-FNB | 215 |  | Retained in primary overall DTA after cohort/dependency audit. | 1 |
| Okasha2024b | 2 | Okasha | 2024 | MOSE | Mixed/unclear FNA-FNB | 184 | Okasha2024-overall-FNBsubset | Excluded: FNB subset nested within the 215-participant Okasha2024 cohort; overall cohort dataset retained. | 0 |
| Ratnaraj2018 | 1 | Ratnaraj | 2018 | ROSE | Mixed/unclear FNA-FNB | 193 |  | Retained in primary overall DTA after cohort/dependency audit. | 1 |
| Ratnaraj2018 | 2 | Ratnaraj | 2018 | NONE | Mixed/unclear FNA-FNB | 80 |  | Retained in primary overall DTA after cohort/dependency audit. | 1 |
| Rizza2021 | 1 | Rizza | 2021 | MOSE | FNB | 38 | Rizza2021-Gaia2022 | Excluded conservatively: earlier conference report with overlapping authors, setting, design, and sample structure; Gaia2022 full report retained. | 0 |
| Rizza2021 | 2 | Rizza | 2021 | NONE | FNB | 38 | Rizza2021-Gaia2022 | Excluded conservatively: earlier conference report with overlapping authors, setting, design, and sample structure; Gaia2022 full report retained. | 0 |
| So2021 | 1 | So | 2021 | MOSE | FNB | 74 |  | Retained in primary overall DTA after cohort/dependency audit. | 1 |
| Sonthalia, N.2024 | 1 | Sonthalia, N. | 2024 | MOSE | FNB | 48 |  | Retained in primary overall DTA after cohort/dependency audit. | 1 |
| Sonthalia, N.2024 | 2 | Sonthalia, N. | 2024 | NONE | FNB | 48 |  | Retained in primary overall DTA after cohort/dependency audit. | 1 |
| Sundaram2022 | 1 | Sundaram | 2022 | ROSE | Mixed/unclear FNA-FNB | 103 | Sundaram2022-2023 | Excluded from primary overall DTA/NMA: paired/repeated assessment of the same cohort and likely preliminary report; independence cannot be assumed. | 0 |
| Sundaram2022 | 2 | Sundaram | 2022 | MOSE | Mixed/unclear FNA-FNB | 103 | Sundaram2022-2023 | Excluded from primary overall DTA/NMA: paired/repeated assessment of the same cohort and likely preliminary report; independence cannot be assumed. | 0 |
| Sundaram2023 | 1 | Sundaram | 2023 | ROSE | FNB | 155 | Sundaram2022-2023 | Excluded from primary overall DTA/NMA: paired ROSE/MOSE assessment in the same participants; within-person covariance unavailable. | 0 |
| Sundaram2023 | 2 | Sundaram | 2023 | MOSE | FNB | 155 | Sundaram2022-2023 | Excluded from primary overall DTA/NMA: paired ROSE/MOSE assessment in the same participants; within-person covariance unavailable. | 0 |
| Wong2025a | 1 | Wong | 2025 | ROSE | FNB | 65 | Wong2025-repeated | Excluded from primary overall DTA/NMA: repeated within-cohort evaluations of the same 65 participants; not independent diagnostic datasets. | 0 |
| Wong2025b | 2 | Wong | 2025 | MOSE | FNB | 65 | Wong2025-repeated | Excluded from primary overall DTA/NMA: repeated within-cohort evaluations of the same 65 participants; not independent diagnostic datasets. | 0 |
| Wong2025c | 3 | Wong | 2025 | ROSE | FNB | 65 | Wong2025-repeated | Excluded from primary overall DTA/NMA: repeated within-cohort evaluations of the same 65 participants; not independent diagnostic datasets. | 0 |
| Xu2024a | 1 | Xu | 2024 | NONE | FNB | 96 |  | Retained in primary overall DTA after cohort/dependency audit. | 1 |
| Xu2024b | 2 | Xu | 2024 | NONE | FNB | 97 |  | Retained in primary overall DTA after cohort/dependency audit. | 1 |
| Zhang2020 | 1 | Zhang | 2020 | ROSE | FNA | 60 |  | Retained in primary overall DTA after cohort/dependency audit. | 1 |
| Zhang2020 | 2 | Zhang | 2020 | NONE | FNA | 64 |  | Retained in primary overall DTA after cohort/dependency audit. | 1 |
| Zhao2024a | 1 | Zhao | 2024 | ROSE | FNA | 52 | Li2023-Zhao2024 | Excluded: exact 2x2 duplicate of Li2023; journal report retained. | 0 |
| Zhao2024b | 2 | Zhao | 2024 | ROSE | FNA | 45 | Li2023-Zhao2024 | Excluded: exact 2x2 duplicate of Li2023; journal report retained. | 0 |
| Zhao2024c | 3 | Zhao | 2024 | NONE | FNA | 91 | Li2023-Zhao2024 | Excluded: exact 2x2 duplicate of Li2023; journal report retained. | 0 |

Supplementary Table S6. Study-level independent direct comparisons.

| Study | Comparison | Needle | OR | 95% CI | log OR | SE |
| --- | --- | --- | --- | --- | --- | --- |
| Cai et al., 2021 | ROSE vs NONE | FNA | 2.581 | 0.575-11.576 | 0.948 | 0.766 |
| Li et al., 2023 | ROSE vs NONE | FNA | 10.133 | 2.259-45.453 | 2.316 | 0.766 |
| Lin et al., 2023 | ROSE vs NONE | FNA | 5.072 | 1.731-14.857 | 1.624 | 0.548 |
| Zhang et al., 2020 | ROSE vs NONE | FNA | 10.926 | 1.353-88.207 | 2.391 | 1.066 |
| Crino et al., 2021 | ROSE vs NONE | FNB | 0.705 | 0.309-1.607 | -0.350 | 0.420 |
| Dbouk et al., 2025 | ROSE vs NONE | FNB | 1.068 | 0.222-5.126 | 0.066 | 0.800 |
| De Lusong et al., 2024 | ROSE vs NONE | FNB | 1.000 | 0.060-16.578 | 0.000 | 1.433 |
| Fabbri et al., 2017 | ROSE vs NONE | FNB | 1.587 | 0.746-3.373 | 0.462 | 0.385 |
| Amalou et al., 2025 | MOSE vs NONE | FNB | 1.597 | 0.612-4.168 | 0.468 | 0.490 |
| Gaia et al., 2022 | MOSE vs NONE | FNB | 0.776 | 0.192-3.147 | -0.253 | 0.714 |
| Mangiavillano et al., 2023 | MOSE vs NONE | FNB | 1.253 | 0.654-2.402 | 0.226 | 0.332 |
| Sonthalia et al., 2024 | MOSE vs NONE | FNB | 2.091 | 0.364-11.996 | 0.738 | 0.891 |
| Comas et al., 2024 | MOSE vs ROSE | FNB | 0.416 | 0.020-8.868 | -0.876 | 1.560 |
| Ishikawa et al., 2022 | MOSE vs ROSE | FNB | 4.500 | 1.656-12.229 | 1.504 | 0.510 |
| Ogura et al., 2024 | MOSE vs ROSE | FNB | 0.667 | 0.109-4.094 | -0.405 | 0.926 |

Supplementary Figure Legends

Supplementary Figure S1. Randomized-comparison-only direct meta-analysis for correct versus incorrect diagnosis. Panel A shows ROSE versus NONE in FNB studies; Panel B shows MOSE versus NONE in FNB studies; Panel C shows MOSE versus ROSE in FNB studies. A single eligible randomized comparison is shown without pooling when meta-analysis is not informative.

Supplementary Figure S2. Restricted analysis of explicitly standardized MOSE definitions. Panel A shows standardized MOSE versus NONE in FNB studies; Panel B shows standardized MOSE versus ROSE in FNB studies. Standardized MOSE indicates that an explicit score or core-length threshold was confirmed in the available report.

Supplementary Figure S3. Full 46-dataset coupled forest plot of sensitivity and specificity after the cohort-overlap/dependency audit. Study-level exact binomial confidence intervals are shown; the bivariate pooled estimates are displayed at the bottom.

Supplementary Figure S4. Study-level component diagnostic outcomes. Random-effects pooled absolute differences in sensitivity and specificity are shown for the four independent direct-comparison contrasts.

Supplementary Figure S5. Certainty-of-evidence profile for the revised synthesis. The figure summarizes certainty judgments for overall DTA, direct comparative estimates, and the exploratory network meta-analysis after considering risk of bias, inconsistency, indirectness, imprecision, and small-study effects.
